# Optimization of Osmotic Dehydration of Tomatoes in Solutions of Non-Conventional Sweeteners by Response Surface Methodology and Desirability Approach

**DOI:** 10.3390/foods9101393

**Published:** 2020-10-01

**Authors:** Maria C. Giannakourou, Andriana E. Lazou, Efimia K. Dermesonlouoglou

**Affiliations:** 1 Laboratory of Chemistry, Analysis & Design of Food Processes, Department of Food Science and Technology, School of Food Sciences, University of West Attica, Agiou Spyridonos, 12243 Aigaleo, Athens, Greece; alazou@uniwa.gr; 2 Laboratory of Food Chemistry and Technology, School of Chemical Engineering, National Technical University of Athens, 5, Iroon Polytechniou, 15780 Zografou, Athens, Greece; efider@chemeng.ntua.gr

**Keywords:** *S. lycopersicum*, osmosis, candying, alternative sugars, Response Surface Methodology, color, texture

## Abstract

The osmotic dehydration (OD) of tomatoes in solutions of alternative sweeteners was investigated using Response Surface Methodology (RSM), while selected desirability functions were implemented in order to define the optimum process parameters (temperature/duration of osmotic treatment, osmotic solution composition and concentration). Mass exchange, color and texture were measured during the process. Changes in color occurred rapidly at the beginning of the process, while firmness was significantly increased, indicating that OD processing led to tomato texture improvement. Color and firmness changes were adequately modeled using a polynomial model. RSM coupled with desirability functions was applied to optimize OD procedure in terms of color retention and maximum solid gain, a requirement for candied products. A maximum desirability was obtained by incorporating oligofructose into the osmotic solution, at relatively short treatment times. Results were validated and sensory analysis was conducted at the optimized conditions to assess samples’ organoleptic acceptance.

## 1. Introduction

The tomato (*Solanum lycorpesicum*) is one of the most popular agricultural commodities in the world, mainly due to the natural occurrence of functional substances such as lycopene, beta-carotene, vitamin C, etc., leading to decreased risk of chronic diseases such as cancer and cardiovascular disease [1]. Among the different processed tomato products, dehydrated tomatoes are less appreciated [2], probably due to the detrimental effect of hot air drying on the quality of final dried products. Candied tomato is a dried tomato product of specific interest for certain regions of Greece. Candying is actually an osmotic dehydration process, deriving a final product of reduced water activity and enhanced sweetness, due to the desirable immersion of selected carbohydrates. These products also possess a significantly longer shelf-life and sensory attributes that are quite different from the raw material [3]. Nevertheless, besides being an intense and quality detrimental procedure due to the high temperatures imposed [4,5], the traditional candying process is time-consuming and cannot be easily standardized [6]. Additionally, the increased sucrose content of the final product is another disadvantage of the conventional candying process, taking into account the current consumer requirements for a healthy and balanced diet [7]. Therefore, innovative candied fruits could be designed based on alternative, healthier carbohydrates that may substitute sugar without downgrading product quality [7,8].

Osmotic dehydration (OD) of tomato has been widely investigated [9,10,11,12], especially as a pre-treatment method to lower product water activity. The OD process is based on the developed osmotic pressure gradient, when the food is immersed in a hypertonic solution; the main mass transfer phenomena that are observed through the semipermeable membrane involve the diffusion of water out and solutes inside the product [13,14,15,16,17]. The main benefits of this process include the use of mild conditions, as well as the selective impregnation of the osmosed tissue with the compounds of the OD solution (carbohydrates, salts, etc.), producing a modified commodity with the desired attributes [18,19]. Nonetheless, osmotic dehydration cannot generate shelf stable products by itself; instead, intermediate moisture foods are produced, that need a complimentary preservation process to further reduce water activity and increase shelf life. In this context, osmotic dehydration is combined either with high-temperature treatments, such as air-drying [20,21,22,23,24], or with preservation at low temperatures (osmodehydrofreezing) [25,26].

As thoroughly studied in the recent literature, osmotic dehydration is mainly affected by processing factors, such as the type and concentration of the osmotic medium, temperature, OD solution/product mass ratio, physicochemical properties of the tissue to be treated application of agitation, use of complimentary techniques to facilitate mass transfer [12,27,28,29,30,31,32,33] One of the prevailing parameters is the selection of carbohydrates, salts and/or other compounds and their concentration in the osmotic solution. Until recently, common sugars were the most widely applied [34]. The use of ternary solutions (complex solutions, including both carbohydrates and salts) and especially the substitution of sucrose with other sweeteners is less frequently addressed in literature, but is a topic of increasing interest, in order to produce foods with a less sweet taste that do not greatly deviate from the sensory attributes of the initial raw tissue. 

The most important requirements for an osmotic agent are related to its molecular weight, its solubility and its sensory properties. Carbohydrates with relatively low molecular weight such as sucrose, glucose, and fructose are found to enhance the solid uptake due to the high diffusion of small molecules. Other sweeteners such as maltodextrins, fruit juices, and stevia are also used as osmotic agents for OD, which could be preferred by people with health issues, such as diabetes, obesity, dental caries, etc. [23]. The increasing interest in the natural sweetener steviol glucoside is related to it being a plant extract, calorie-free and with sweetness much higher that the sucrose equivalent, making it an ideal choice for several food and beverage applications [35]. Osmotic agents including stevia-based sweeteners have been recently reported for plant tissue treatments [36,37,38]. Isomaltulose, often mentioned with the commercial name of palatinose (6-O-α-D glucopyranosyl-D-fructose) is a reducing disaccharide which is naturally present in honey and sugar cane juice, with a taste similar to sucrose and about 42% of its sweetness [36,39], making it a potential sucrose substitute [40]. Fructooligosaccharides (FOS) are another group of potential osmotic agents, frequently applied in recent research regarding OD of fruits and vegetables [3,36,41,42]. They are non-digestible oligosaccharides, having the properties of dietary fibers and prebiotic, that help in enhancing the growth of beneficial gut bacteria and calcium absorption [36]. 

OD parameters can be manipulated so as to attain the desirable characteristics of the treated tissue, by systematically measuring and controlling water activity, the impregnation of specific solutes (through the measured solid gain) and the sensory profile of the osmodehydrated product. The application of an appropriate experimental design for process design, optimization and the production of high quality products is of great importance. In this context, Response Surface Methodology (RSM) can be used to develop, improve, and finally optimize the OD procedure. A very interesting application of the RSM technique is related to the possibility of meeting multiple criteria and thus, being able to optimize multiple responses of the OD process. This calculating procedure, known as the ‘desirability approach’, coupled with the traditional RSM steps, is rarely applied in OD studies, although its implementation could give valuable information on the appropriate conditions for formulating a product in a desired direction. Few applications of this optimizing technique are available in the recent literature [12,43,44]; the RSM, coupled with desirability functions, was applied to optimize the OD process [12]. 

The main scope of this research was to study the osmotic dehydration in complex osmotic solution of alternative carbohydrates, besides sucrose, for obtaining dehydrated tomatoes, as a preliminary step in the candying process. The current research is focused on implementing the principles of Response Surface Methodology (RSM) in order to define the effect of the independent variables of the OD process on the indices selected and propose an optimized OD treatment, based on multiple responses using the desirability approach. For this purpose, an appropriate experimental design is carried out, according to which, color and firmness change, as well as mass exchange indices during the OD process were calculated. As published in [12], the quality attributes cannot be excluded from desirability constraints, in order to practically obtain an acceptable final product that meets the necessary quality specifications. It is worth stressing that the practical aspect of this work involves the production of intermediate moisture tomatoes, that would be submitted to a further candying step (of reduced intensity and duration, compared to the one step traditional candying process) to produce candied tomatoes of increased shelf life and standard quality.

## 2. Materials and Methods 

### 2.1. Pretreatments and Osmotic Dehydration (OD) Process

Lobello type tomatoes (*Solanum lycorpesicum*), of a cylindrical shape and a mean diameter of 30 ± 10 mm, were purchased from a local market (Spata, Athens), transported to the laboratory and stored at 4.0 ± 0.5 °C for a maximum of one day. Tomatoes were sorted to ensure that they were uniform in maturity and size, and they were washed with tap water in order to remove the spores. Then, a 60 s blanching step was carried out, in order to remove the peel, before soaking tomato samples in a solution of 1.5% calcium chloride (CaCl_2_) (weight ratio of tomato sample to solution 1:2) at room temperature (23 ± 1 °C) for 8 h [45,46], aiming at a maximum texture reinforcement. The water content of the raw material was calculated as 93.21 ± 1.07 g/100 g dry weight, the a_w_ was 0.9931 ± 0.0070 and the total soluble solids were 7.0 ± 0.7 °Brix (mean and standard deviation out from five replicates), measured in the pulp, produced after manually grinding whole tomato fruit until being fully homogenized. 

Osmotic solutions were prepared by dissolving in tap water conventional (sucrose, S) and non-conventional sweeteners, such as oligofructose and mixtures commercially available for industrial use in pastry (Excellent Stevia, Egaleo, Greece), containing palatinose (isomaltulose)/steviol glucosides (IS) and polydextrose/sucralose/steviol glucosides (PS). Five different types of osmotic solutions were tested, containing oligofructose, coded as ‘O’; a mixture of sucrose and oligofructose (in a ratio of 1:1; coded as ‘SO’), and mixtures based on the aforementioned commercial sweeteners, namely a mixture of (IS) + oligofructose (in a ratio of 1:1, coded as ‘ISO’) and a mixture of (PS) + oligofructose (in a ratio of 1:1, coded as ‘PSO’). Solution concentration expressed as water soluble solids (°Brix) was measured by hand refractometer at 25 °C (ATAGO hand refractometer, Japan).

Tomatoes were osmotically processed at temperatures (T) of 75, 85 and 95 °C for times up to 180 min and osmotic solute concentrations (C) of 65, 70 and 75°Brix (weight ratio of tomato sample to osmotic solution 10:1), in conditions adopted from a previous work [45]. Pre-weighed tomato samples were immersed in the osmotic solution in beakers thermo-stated in water baths. At the selected times, samples were removed from the osmotic solution, rinsed with water, wiped carefully with absorbent paper, and weighed. Experiments were performed in triplicate.

### 2.2. Physico-Chemical and Quality Parameters

#### 2.2.1. Mass Transfer

Mass transfer parameters were calculated in terms of water loss (WL), Equation (1), and solid gain (SG), Equation (2), and their change during OD was discussed in detail in [45]. Water activity (a_w_) was also monitored during the process (Aqua LAB 4TEV, METER Group, Inc., Washington, DC, USA).

(1) $$WL=\frac{\left(M_{0}-m_{0}\right)-\left(M-m\right)}{m_{0}}$$

(2) $$SG=\frac{\left(m-m_{0}\right)}{m_{0}}$$

where *M*_0_ is the initial mass of fresh material before the osmotic treatment, *M* is the mass of tomato samples after time t of osmotic treatment, *m* is the dry mass of tomato after time *t* of osmotic treatment and *m*_0_ is the dry mass of fresh material.

#### 2.2.2. Color

Tomato color was expressed by the values, *L*, *a* and *b*, and the values for total color change, calculated by the following equation, E

(3) $$\Delta E=\sqrt{{\left(L-L_{0}\right)}^{2}+{\left(a-a_{0}\right)}^{2}+{\left(b-b_{0}\right)}^{2}}$$

where the index ‘0′ denotes time zero measurements. Color data are provided as CIE Lab coordinates (Model H-CT, SUGA Test Instruments, Japan), which defines color in a three dimensional space. *L* indicates lightness, taking values within the range between 0 (black) and 100 (white), and “*a*” and “*b*” are green-red and blue-yellow coordinates, respectively. Moreover, *a* takes positive values for reddish colors and negative values for greenish ones, whereas *b* takes positive values for yellowish colors and negative values for bluish ones [47].

#### 2.2.3. Texture

Texture measurements were conducted by means of a texture analyzer (TA-XT2i of Stable Micro Systems, Godalming, UK), and a Texture Profile Analysis (TPA) test was carried out using tomatoes of cylindrical shape (3 replicates). The test was performed on a non-lubricated flat platform using a 60-mm cylindrical compression probe and a 25 Kg load cell. Samples were twice compressed using a fixed rate (1 mm/s) at 50% deformation. Texture characteristics such as firmness, adhesiveness, cohesiveness, springiness, and chewiness were calculated [48,49]. Amongst the different parameters calculated from texture measurements, firmness, F_max_, was found to be the more representative, and was further analyzed to draw some reliable conclusions. Firmness values are expressed in Newtons (N) and denote the maximum force (N) necessary to compress the tomato samples.

#### 2.2.4. Sensory Analysis

Trained sensory panelists, after three extended training sessions, rated the main sensory properties of the OD-processed tomatoes (representative samples corresponding to OD optimized conditions, as estimated by RSM—see Section 2.3). Scores were given for each parameter separately on a 1–9 intensity scale (1, the lowest intensity–9, the highest intensity): red color, shrinkage, texture, firmness, adhesiveness, cohesiveness, springiness, chewiness, sweetness, bitterness, aftertaste, as well as on a 1–9 intensity scale (1, the lowest quality score–9, the highest quality score): appearance, texture, flavor/taste and total sensory quality.

### 2.3. Experimental Design and Statistical Analysis 

Response Surface Methodology (RSM) was selected to estimate the main effects of the process variables on mass transfer and quality-related variables during the osmotic dehydration of tomatoes. Three parameters, namely temperature (X_1_), osmotic solution concentration (X_2_) and treatment time (X_3_), were selected as the most important independent factors based on literature reports and preliminary experiments. The range of OD time (ranging between 30–90 min), as well as the range of temperature, and the concentrations of the different osmotic agents used were decided based also on the findings of our previous work [45]. 

A second order polynomial model was used for the determination of the factor interactions during OD. The second order model is usually sufficient for the optimum region, as third order and higher effects are rarely important. This model describes the response variables Y (a_w_, water loss—WL, solid gain—SG, color—ΔE, texture—F_max_) as a function of the factor variables *X*_i_ (i assuming values from 1 to 3) (Equation (4)), for the all osmotic solutions used [50].

(4) $$Y=a_{0}+\sum^{\;}a_{i}X_{i}+\sum^{\;}a_{ii}X_{Ι}^{2}+\sum^{\;}a_{ij}X_{i}X_{j}$$

where α_ο_ is the constant, α_i_ is the linear, α_ii_ is the quadratic and α_ij_ is the interaction effects of the factors. The model contains p = [(3 + 1)(3 + 2)]/2 = 10 regression parameters that include coefficients for the main effects (a_1,_ a_2,_ a_3_), coefficients for quadratic main effects (a_11,_ a_22,_ a_33_) and coefficients for two factor interaction effects (a_ij_). Regarding the interpretation of the coefficients in Equation (4), their positive or negative value is a sign of a promoting or an opposing effect on the response, respectively. After developing the polynomial models, analysis of variance (ANOVA) was implemented to assess how well the model describes the data, and p-value criteria were used to assess the statistical significance. Since this study requires three evenly spaced levels, the Box–Behnken design was used (Table 1). The Box–Behnken design is an efficient alternative to the central composite design, since fewer runs are necessary [51]. 

For better depicting the individual (linear or quadratic) and combined effects of the independent variables on the quality indices of tomato samples measured, 3D plots were constructed where the two independent variables were allowed to vary within the range tested, while the third variable was fixed at the central point of the experimental design. 

Regarding Response Surface Methodology, the optimization of multiple responses was performed by using the desirability functions proposed by [52,53,54]. Under this approach, each ith response is assigned a desirability function, d_i_, where the value of d_i_ varies between 0 and 1. This function is defined in a different way, based on the objective of the response. If the target of the procedure followed (here OD of tomatoes) is to maximize the specific response, as in the case of SG of tomatoes (that are supposed to be further processed for candied products), then it is defined as follows:

(5) $$d_{1,i}=\{\begin{array}{c} 0\;\;\;\;\;\;\;\;\;\;\;\;\;\;\;\;\;\;\;y_{i}<L\\ \left(\frac{y_{i}-L}{U-L}\right)\;\;\;\;\;\;\;\;\;\;\;\;\;L\leq y_{i}\leq U\\ 1\;\;\;\;\;\;\;\;\;\;\;\;\;\;\;\;\;\;\;y_{i}>U \end{array}$$

where *U* represents the target value of the ith response (here equals SG_max_), and *L* represents the acceptable lower limit value for this response (here equals to 0).

If the response is to be minimized, as in the case when the response is color change (expressed by Equation (3)), d_i_ is defined as follows:

(6) $$d_{2,i}=\{\begin{array}{c} 1\;\;\;\;\;\;\;\;\;\;\;\;\;\;\;\;\;\;\;y_{i}<L\\ \left(\frac{U-y_{i}}{U-L}\right)\;\;\;\;\;\;\;\;\;\;\;\;\;L\leq y_{i}\leq U\\ 0\;\;\;\;\;\;\;\;\;\;\;\;\;\;\;\;\;\;\;y_{i}>U \end{array}$$

where *L* and *U* are the lower and upper boundary of the independent variables, respectively. In our case, when desirability was described in terms of color change ΔE, *L* is equal to 0 and *U* is set to the fixed value of 9, determined by a preliminary sensory test as the limit of acceptability, in terms of color modification. 

Once a desirability function is defined for each of the *i* responses for the criteria chosen (SG maximization and color change minimization), an overall desirability function is obtained as follows:

(7) $$d_{overall,i}={\left(d_{1,i}^{r_{1}}\cdot d_{2,i}^{r_{2}}\right)}^{1/\left(r_{1}+r_{2}\right)}$$

where *r*_1_ and *r*_2_ represent the importance of each response. The greater the value of *r*_i_, the more important the response with respect to the other responses. The objective is to find the settings (here process parameters, namely OD temperature, OD concentration and immersion time) that return the maximum value of d_overall_. The choice of desirability criteria does not limit the implementation of the methodology; for example, instead of SG and color change factors, one could alternatively select a_w_ decrease and firmness retention instead, if the goal was the production of an intermediate moisture product (IMF) of extended shelf life.

As far as statistical analysis of ΔE and firmness values is concerned, the experimental data were analyzed using the tool of the analysis of variance (ANOVA)(STATISTICA 12.0), and significant differences were assessed using Tukey’s post hoc HSD test. The analysis of Box–Behnken design and parameter optimization through the desirability functions methodology was implemented, using the Minitab^®^ (DOE-Response Surface application). 

## 3. Results and Discussion

### 3.1. Process Parameters’ Effect on Color Characteristics during Osmotic Dehydration (OD)

The effect of process parameters on tomato color was investigated during the OD process. Tomato color parameters measured were in accordance with those reported in literature [12,55]. Changes in color parameters (L, a, b) occurred more rapidly at the beginning of the process (before 90–120 min), when WL and SG were faster according to the kinetic study presented in [45], an observation that agrees with findings in [56]. It was observed that OD treatment modified the color of fresh tomato leading to lower L (decreased lightness or whiteness) and b (decrease in yellowness) values and higher a (increase in redness) values for all osmotic solutes (S, O, SO, PSO, ISO) (data not shown). The reduction in L observed could be attributed to the opacity increase of samples as a result of the shrinkage occurring during OD processing [57]. The behavior of these parameters could be attributed to liquid phase concentration occurring during OD processing [56,58]. Although not measured in this work, browning of bright red color of tomatoes, especially after being treated at elevated temperatures (such as at 95 °C), can be attributed to both the Maillard reaction and degradation of lycopene [59,60]. 

**Figure 1:**
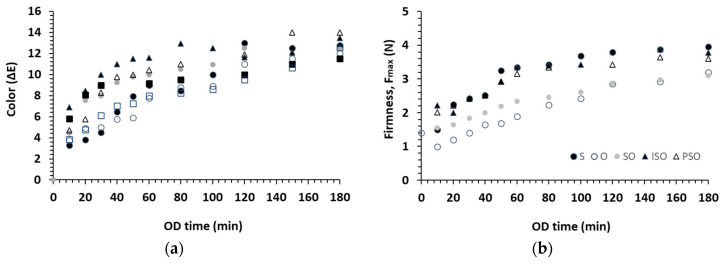
(**a**) Total color (ΔE) and (**b**) firmness (F_max_) changes during OD of tomatoes using different sweetener (S, O, SO, PSO, ISO) in the osmotic solution; OD solute concentration: 70°Brix; OD temperature: 85 °C. Points represent average values from the three measurements.

To express the total color change of tomato samples during OD processing, the ΔE value (Equation (1)) was used. In Figure 1a, ΔΕ changes of representative tomato samples during OD are presented for all osmotic solutes (representatively for the central point of OD solute concentration (C): 70°Brix and OD temperature (T): 85 °C). Color change increased immediately after OD application (from initial times), and during progress (>10 min up to the end of OD processing). Similar charts are constructed for all set of experimental conditions, showing similar trends (data not shown). At the same time, besides instrumental measurements, the sensory evaluation of the final color of the osmosed tomato samples was simultaneously conducted in order to determine the limit of acceptability (ΔΕ_max_ value, a necessary information for RSM implementation).

### 3.2. Process Parameters’ Effect on Texture Characteristics during Osmotic Dehydration (OD)

Tomato firmness has been reported to be one of the most important tomato quality parameters. Tomatoes exhibiting lower firmness values are characterized by lower quality [61]. OD-processed tomato samples presented increased firmness compared to the fresh ones, indicating that OD processing led to tomato texture improvement [25,37,38,62,63]. OD processing induces a greater firmness by filling the pores with the osmotic solution, thus the OD-processed samples present a more compact and less deformed tissue [64]. 

The increase in the adhesiveness of OD-processed samples has been previously reported [63]. In [63], the authors observed that adhesiveness of OD-processed plum increased. The adhesiveness assumed higher values in processed plum samples in glucose solution compared to those samples processed in a sucrose solution (30 min osmotic process). This increase could be related to the distribution of sugar content in the sample tissue—more specifically, the adherence of sucrose content on the surface of samples, leading at the same time to the increase in firmness. The authors in [7] studied candied fruits (pineapple, orange-peel, citron) developed by substituting the sucrose and glucose with fructose, sorbitol, maltitol, fructooligosaccharides (FOS) and galactooligosaccharides (GOS). They concluded that each osmotic solute significantly affected the texture of the candied fruits, but these changes were dependent on the fruits. For example, firmness and fracturability increased for all the fruits apart from pineapple, when FOS was used. The values of firmness for all candied fruits OD-processed with the alternative osmotic solutes were higher than the respective values of commercially candied fruits (sucrose). Springiness, cohesiveness and chewiness were higher in orange peel, independently of the osmotic solute used apart from FOS. In the present study, SO-, O- and S-processed tomato samples presented similar adhesiveness values to the respective values of fresh tomato samples, followed by PSO- and ISO-processed samples. An increase in solute concentration as well as an increase in temperature (in most cases) caused an increase in adhesiveness. OD processing time caused the increase in tomato cohesiveness, springiness and chewiness. The increase in cohesiveness, springiness and chewiness for OD-processed samples has been also reported in several studies [62,63]. In [63], authors observed that the OD-processed plum samples in glucose and sucrose solutions (e.g., 60 min OD time) presented increased values of cohesiveness, springiness and chewiness. Cohesiveness is strongly correlated with firmness; chewiness is strongly correlated with firmness, cohesiveness and springiness [61]. In [65], it was reported that cohesiveness, springiness and chewiness demonstrated similar three-phase behavior with the dependence on moisture content of dehydrated apple samples. 

To express the textural changes of tomato samples during OD processing, the firmness value was selected as the representative texture parameter. In Figure 1b, the firmness of tomato samples during OD processing is presented for all osmotic solutes (representatively, for the central point of OD solute concentration (C): 70°Brix and OD temperature (T): 85 °C). The firmness showed similar behavior with color change during OD processing; it increased immediately after OD application (from initial times) and during progress (>10 min up to the end of OD processing). 

### 3.3. Determination of Quality Factor Interactions during Osmotic Dehydration (OD)

The coefficients calculated from the second order polynomial model (Equation (4)) regarding color (ΔE) and texture (firmness) of OD-processed tomatoes are presented in Table 2 and Table 3, respectively. Similar polynomial equations referring to WL, SG and a_w_ change were presented and commented on in detail in [45].

According to the regression coefficients, ΔE values are influenced mostly by solute concentration (a_2_), as shown by the higher values of the corresponding factors (Table 2), and much less by temperature (a_1_) and time duration (a_3_). Temperature and solute concentration have a significant effect on ΔE values for SO and ISO samples. No synergistic effects, interactions between solute concentration and time duration, and interactions between temperature and time duration were detected. Temperature and time duration have a positive effect on ΔE (no significant effects), and sweetener concentration have a negative effect on ΔE for PSO (no significant effect). 

The firmness values were mostly affected by solute concentration (a_1_) and temperature (a_2_); the corresponding factors were much lower compared to the ΔE values for all sweeteners. Time duration (a_3_) seemed not to affect the firmness values (the lowest corresponding factors) (Table 3). Temperature has a significant effect on firmness for SO, ISO and PSO. Interactions between sweetener concentration with temperature were found to have a significant effect on firmness for O, PSO and ISO. 

**Figure 2:**
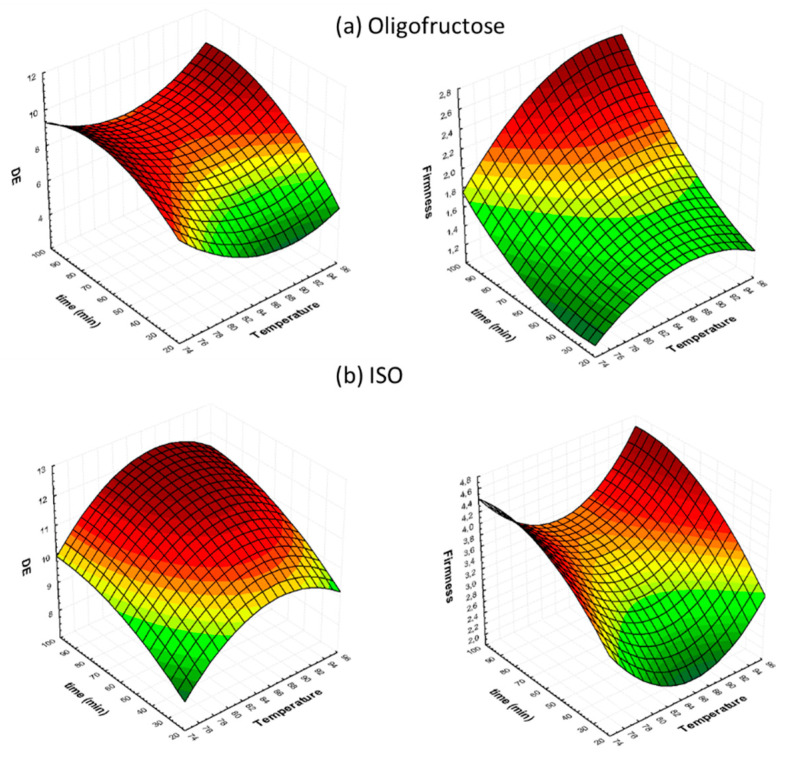
Three-dimensional Response Surface graphs for color change (ΔE) and firmness, as a function of process duration and temperature, at a fixed OD concentration of 70°Brix, in the case of: (**a**) O and (**b**) ISO osmotic solutions.

**Figure 3:**
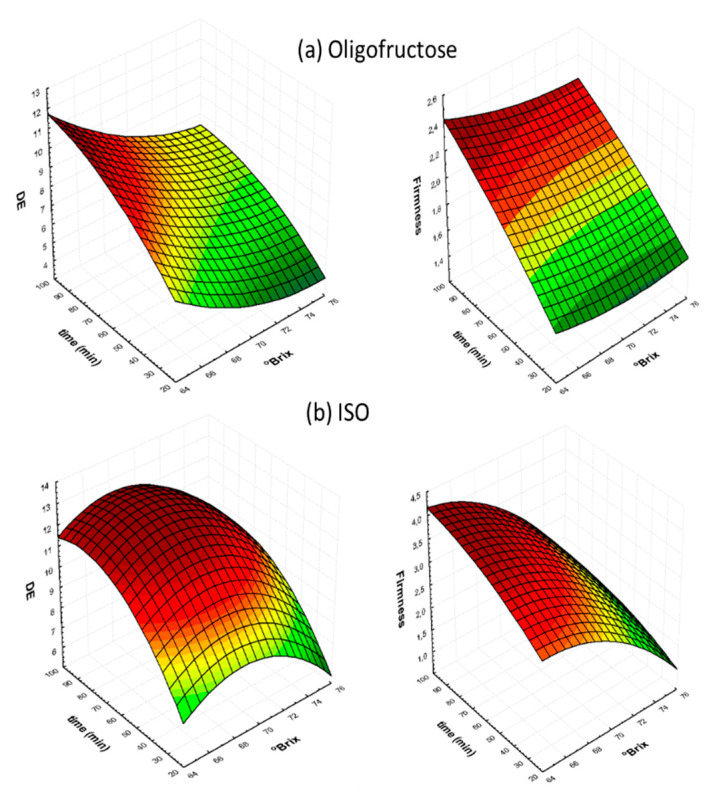
Three-dimensional Response Surface graphs for color change (ΔE) and firmness, as a function of process duration and OD solution concentration, at a fixed temperature of 85 °C, in the case of: (**a**) O and (**b**) ISO osmotic solutions.

**Figure 4:**
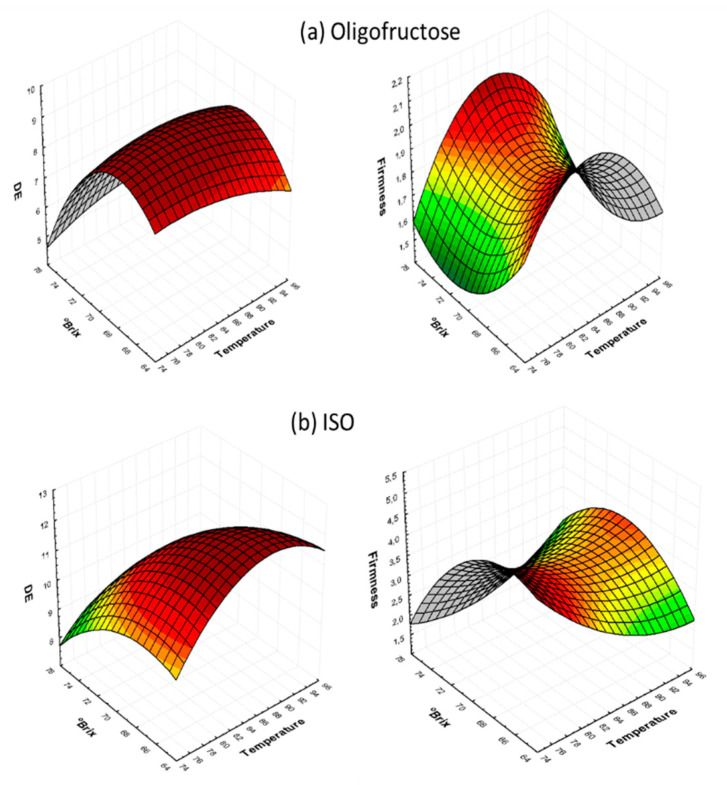
Three-dimensional Response Surface graphs for color change (ΔE) and firmness, as a function of process temperature and OD solution concentration, at a fixed time of 60 min, in the case of: (**a**) O and (**b**) ISO osmotic solutions.

In Figure 2, Figure 3 and Figure 4, the 3D response surface graphs illustrate the combined effect of the independent process variables on the quality parameters studied (indicatively, for O and ISO osmotic solutions), These figures provide useful information about the behavior of the system within the experimental design of Table 1. These plots are constructed, allowing for two variables to change, while the third independent variable assumes a constant value. Figure 2 shows the effect of treatment time and temperature (while keeping OD solution concentration constant at the central point) on ΔE and firmness. As expected, ΔE and firmness exhibit a significant increase during OD at all temperatures tested. Based on these graphs, several combinations of independent variables can be identified by interpolation to obtain tomato samples with desirable ΔE and firmness values. 

Regarding the effect on ΔE and firmness, Figure 3 shows the effect of osmotic solute concentration and treatment time (while keeping OD temperature constant), and one can observe the mild influence of osmotic concentration, whereas OD time significantly affected quality changes of OD samples (the same trend was observed for all different osmotic solutes). 

Figure 4 shows the combined effect of OD solution concentration and temperature (at the fixed time of 60 min, central point in Table 1). In the case of ISO, ΔE increases as both OD solution concentration and temperature increase, whereas for oligofructose-treated samples, the effect of OD concentration is more important. Similar 3D plots to those of Figure 2, Figure 3 and Figure 4 were constructed for all OD solutes (data not shown), which revealed similar trends to the ones discussed for O and ISO samples.

It could be concluded that both the color and texture changes of OD-processed tomato samples were strongly dependent on processing parameters for the different osmotic solutes studied.

### 3.4. Optimization of Process Conditions, Based on Mass Transfer and Quality Requirements

**Figure 5:**
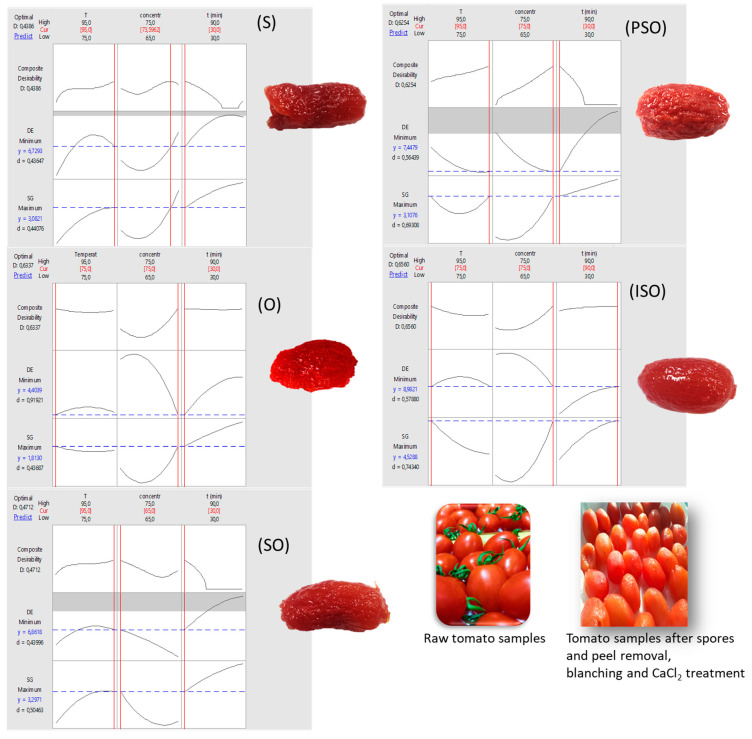
Desirability plots of variables for the different osmotic agents used (coded inside the parenthesis) and illustrations of tomato samples at the optimized conditions. Photos of the raw material, as well as for samples after the OD pretreatment (including spore and peel removal, blanching and CaCl_2_ treatment) are also provided.

**Figure 6:**
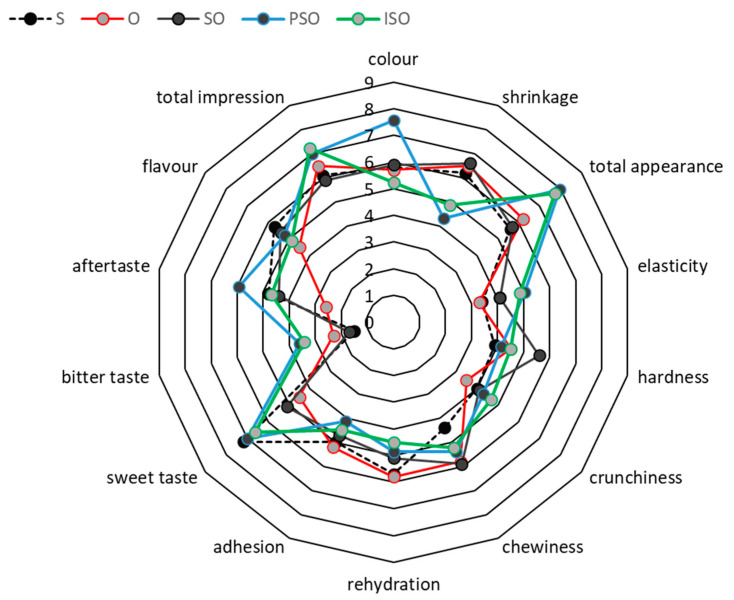
Sensory scores for the attributes on the hedonic scale [OD solute type: S, O, SO, PSO, ISO] at the optimum process conditions, as depicted n Table 4. Average scores (scale 1–9) for color (axis 1), shrinkage (axis 2), total appearance (axis 3), firmness (axis 4), adhesiveness (axis 5), springiness (axis 6), chewiness (axis 7), total texture (axis 8), sweet taste (axis 9), bitter taste (axis 10), aftertaste (axis 11), total flavor/taste (axis 12) and total impression (axis 13).

The desirability function method was implemented to locate the process parameter values that meet the criteria defined, to optimize the dependent variables (SG and color change). The corresponding profiles of composite desirability are depicted in Figure 5. The desired levels for each of the operational conditions (temperature, OD time, °Brix) were selected within the range defined by the experimental procedure (Table 1). The results are shown in Table 4, demonstrating the variability of optimum conditions, depending on the type of osmotic solute used. In the same table (Table 4), estimated values of all important factors, namely a_w_, WL, SG, color change and firmness, are calculated at those optimum conditions, based on the second order models developed. A significant observation is that SG is maximized with the use of oligofructose in the osmotic solutions, retaining to an acceptable extent the initial color of tomato samples (cases O, OS, ISO and PSO), giving desirability values ≥ 0.6 (with the exception of SO). In the same sample categories, as shown in Table 4, the firmness of samples is slightly increased (at the optimum conditions), and this mild modification is appreciated when further processes (e.g., air drying) are expected to be performed for candying purposes. It can be also observed that high OD concentrations and short treatment times (around 30 min) provide the best results, regarding the criteria set. The regression models and the results presented in Table 4 were validated by performing experiments at the optimum predicted conditions for each of the different osmotic agents used. The conditions were experimentally verified with a deviation of +5.0% compared to the predicted values of a_w_, WL SG, ΔE and firmness. In Figure 5, photos of tomato samples are shown, to have a more realistic illustration of their appearance, at the optimized conditions. Moreover, a sensory evaluation was performed at the optimized conditions, where the organoleptic quality of tomato samples is described in terms of appearance, texture, taste / flavor and total impression. The intensity scores for the attributes on the hedonic scale for all samples are presented in Figure 6. In this figure, average scores (scale 1–9) for color (axis 1), shrinkage (axis 2), total appearance (axis 3), firmness (axis 4), adhesiveness (axis 5), springiness (axis 6), chewiness (axis 7), total texture (axis 8), sweet taste (axis 9), bitter taste (axis 10), aftertaste (axis 11), total flavor/taste (axis 12) and total impression (axis 13) are demonstrated. 

Samples that were OD processed with sucrose solution had the highest scores for sweetness, whereas samples that were OD processed with oligofructose presented much lower scores. A general observation was that the samples processed with polydextrose-sucralose-steviol glucosides (PSO) presented a sweet taste. This could be a result of the combination of sucralose (600 times sweeter than sucrose) and steviol glucosides (300 times sweeter than sucrose), while polydextrose does not present sweetness [66,67]. 

As far as the total sensory quality scores are concerned, PSO and ISO samples were rated with the highest scores, followed by SO and O. Regarding S-processed tomato samples, they were characterized by red to brown color, higher shrinkage and increased firmness, springiness and chewiness, and sweet taste. On the other hand, O, SO, PSO, ISO processed samples were defined as dark red colored (probably due to the products of Maillard reactions, as already discussed in §3.1), with milder texture characteristics similar to those of the fresh tomatoes, and a pleasant, sweet taste. In any case, samples treated with non-conventional osmotic agents were judged as ‘pleasant and acceptable’, obtaining scores slightly higher than those of S-treated samples. It is worth noting that there was no significant difference in total acceptability scores for the different alternative sweeteners applied, leading to a preliminary promising finding that sucrose could be effectively replaced in the traditional tomato candying process. It has been reported that the most important sensory characteristics affecting the consumer acceptance of OD-processed candied fruits are the flavor/taste and texture characteristics depending on the osmotic solute used, as well as the raw material (fruit) [7,68]. From a commercial point of view, it would be necessary to implement a wide consumer’s trial to assess the palatability and acceptance of the different osmodehydrated products.

## 4. Conclusions

The process conditions of the osmotic pretreatment of tomato samples with alternative osmotic solutes can significantly affect quality characteristics (such as texture, color, and sensory attributes), before the subsequent drying step of a candying process. The ultimate purpose of this preliminary dewatering step is to produce intermediate moisture tomato products, that would be further processed to candied counterparts, of improved perishability and innovative, pleasant sensory attributes. In addition to the potential of substituting sugar without compromising sensory acceptability, this intermediate processing step (OD) could also assist in reducing the necessary drying time of the subsequent conventional air drying procedure and the required energy for water removal. The implementation of the Box–Behnken experimental design and RSM, coupled with the desirability approach and selected product requirements, led to the definition of optimum conditions of the OD process, for each of the alternative sweeteners used. A second order polynomial model was proven to adequately describe the effect of the most important OD processing factors (temperature, time, OD solution concentration) on color and firmness attributes. The results obtained reveal that tomato samples, treated with oligofructose-containing solutions in a relatively short time exhibit satisfactory high levels of solid gain, with better retained quality attributes, also judged positively for their sensory characteristics. Without questioning the necessity to perform extended sensory trials to assess the consumer’s acceptance, the results from this study could serve as a basis for further investigating the potential total or partial sugar replacement in the traditional tomato candying process.

## Figures and Tables

**Table 1:** Coded values of the treatment variables for all types of osmotic solutes used and Box–Behnken design.

OD Treatment Variables	Temperature (°C)	Concentration (°Brix)	OD Time (min)	X_1_	X_2_	X_3_
High	95	75	90	+1	+1	+1
Center	85	70	60	0	0	0
Low	75	65	30	−1	−1	−1
Standard order	X_1_	X_2_	X_3_			
1	−1	−1	0			
2	+1	−1	0			
3	−1	+1	0			
4	+1	+1	0			
5	−1	0	−1			
6	+1	0	−1			
7	−1	0	+1			
8	+1	0	+1			
9	0	−1	−1			
10	0	+1	−1			
11	0	−1	+1			
12	0	+1	+1			
13	0	0	0			
14	0	0	0			
15	0	0	0			

**Table 2:** Color (ΔE) coefficients for tomato pre-treated with different osmotic solutions, (Equation (4)), based on experimental data of the design shown in Table 1.

Coefficient ^1^	S	O	SO	PSO	ISO
Constant a_0_	197.021	−191.318	−301.734 *	59.033	−260.059 *
Linear					
a_1_	2.823	−0.003	3.975 *	0.347	1.842 *
a_2_	−9.366	5.810	4.069 *	−1.856	5.626 *
a_3_	0.376	0.094	−0.047	0.151	0.128
Quadratic					
a_11_	−0.0178 *	−0.003	−0.012 *	0.0015	−0.007 *
a_22_	0.065	−0.046 *	−0.011	0.018	−0.0036 *
a_33_	−0.0011	−0.001	−0.001*	−0.0005	−0.001
Interaction					
a_12_	0.005	0.006	−0.030 *	−0.009	−0.010
a_23_	−0.002	0.001	0.001	0.0002	0.001
a_13_	−0.0002	−0.001	0.002	−0.0009	−0.001
R^2^	0.799	0.881	0.957	0.906	0.941

^1^$Y=a_{0}+a_{1}X_{1}+a_{2}X_{2}+a_{3}X_{3}+a_{11}X_{1}{}^{2}+a_{22}X_{2}{}^{2}+a_{33}X_{3}{}^{2}+a_{12}X_{1}X_{2}+a_{13}X_{1}X_{3}+a_{23}X_{2}X_{3}$. *X*_1_ = temperature (°C); *X*_2_ = °Brix sweetener concentration; *X*_3_ = time duration (min). * *p*-value < 0.05 Values assigned an asterisk are statistically significant coefficients at a level of 95%.

**Table 3:** Texture (*Firmness, F_max_*) coefficients for tomato pre-treated with different osmotic solutions, (Equation (4)), based on experimental data of the design shown in Table 1.

Coefficient ^1^	S	O	SO	PSO	ISO
Constant a_0_	−54.187	87.773 *	−35.2925	56.895	−8.9811
Linear					
a_1_	−0.5259	−0.4163	0.4087 *	−2.3113 *	−1.82015 *
a_2_	2.2739	−2.0168 *	0.5165	1.2697	2.655 *
a_3_	0.1519	0.0052	0.0078	0.119	0.00596
Quadratic					
a_11_	0.0007	−0.00039	−0.0014	0.00804 *	0.00534
a_22_	−0.0198 *	0.0104 *	−0.0017	−0.0171	−0.02688 *
a_33_	−0.0001	0.00009	−0.0001	−0.00019	−0.0008
Interaction					
a_12_	0.0057	0.00696 *	−0.0027	0.0131 *	0.01229 *
a_23_	−0.0002	0.00015	0.0006 *	0.00033	0.00025
a_13_	−0.0016	−0.00024	−0.0005	−0.0015	0.0009
R^2^	0.867	0.904	0.937	0.868	0.938

^1^$Y=a_{0}+a_{1}X_{1}+a_{2}X_{2}+a_{3}X_{3}+a_{11}X_{1}{}^{2}+a_{22}X_{2}{}^{2}+a_{33}X_{3}{}^{2}+a_{12}X_{1}X_{2}+a_{13}X_{1}X_{3}+a_{23}X_{2}X_{3}$. *X*_1_ = temperature (°C); *X*_2_ = °Brix sweetener concentration; *X*_3_ = time duration (min). * *p*-value < 0.05 Values assigned an asterisk are statistically significant coefficients at a level of 95%.

**Table 4:** Optimum process conditions, estimated by desirability functions, for the different osmotic solutions (based on solid uptake and color retention criteria) and corresponding predicted values of mass transfer and quality parameters.

OD Process Conditions				Predictions					
OD Solute Type	OD Temperature (°C)	OD Solute Concentration (° Brix)	OD Time (min)	*a_w_*	*WL*	*SG*	ΔE	Firmness (N)	Desirability
S	95	73.6	30	0.8343	11.0584	4.31	8.91	2.81	0.439
O	75	75	30	0.982	7.84	1.01	4.41	1.29	0.634
SO	95	65	30	0.955	11.79	3.31	6.87	1.77	0.472
PSO	95	75	30	0.974	9.01	3.09	7.37	2.86	0.625
ISO	75	75	90	0.942	7.29	4.53	8.98	2.50	0.657
